# Harnessing the transformative potential of gamified virtual reality sports to enhance perceived life expectancy in older men at risk of suicide

**DOI:** 10.3389/fpubh.2025.1656261

**Published:** 2025-12-11

**Authors:** Jinjin Zhang, Bingjie Wu, Xiaofen Ding

**Affiliations:** 1 Zhengzhou Technology and Business University, Zhengzhou, China; 2 Henan Vocational Institute of Arts, Zhengzhou, Henan, China; 3 Hunan First Normal University, Changsha, Hunan, China

**Keywords:** older healthcare, mental health, suicidal thoughts, virtual exercises, aging psychology

## Abstract

**Objectives:**

Suicidal vulnerability in older adults represents a growing public health concern, demanding innovative, non-pharmacological interventions that can promote psychological resilience. The present study aimed to examine the effects of gamified virtual reality (VR) sports training on perceived life expectancy among older men identified as being at elevated psychological risk. The study conceptualized perceived life expectancy as an indirect indicator of suicide-related vulnerability, reflecting an individual’s subjective sense of future time perspective and hopefulness.

**Methods:**

A quasi-experimental pretest–posttest design with a non-equivalent control group was used. Participants were community-dwelling older men screened and confirmed to have suicidal ideation through structured clinical interviews conducted by geriatric and mental-health specialists. A total of 110 eligible participants were included. Consequently, participants were assigned to intervention and control groups using a convenience allocation procedure (non-random assignment). The intervention group (*n* = 55) completed a structured 5-week gamified VR-based exercise program (15 sessions), while the control group (*n* = 55) participated in an equivalently timed traditional exercise program matched for intensity, frequency, supervision, and safety procedures. Perceived life expectancy was measured using a validated adapted version of the Robbins scale. Data were analyzed using ANCOVA controlling for baseline scores and prespecified covariates.

**Results:**

Participants in the gamified VR group showed significantly higher post-intervention scores in perceived life expectancy compared to the control group (*F*(1,107) = 796.466, *p* < 0.001, *η*^2^ = 0.882), indicating a large and meaningful intervention effect. Improvements in perceived life expectancy were interpreted as a reduction in psychological vulnerability and an enhanced sense of future orientation.

**Conclusion:**

Gamified VR sports training appears to be an effective and engaging non-pharmacological approach to fostering psychological well-being and hopefulness in older adults at risk of suicide. By integrating physical activation, cognitive stimulation, and immersive engagement, this method offers a scalable and technologically supported pathway for mental health promotion in older populations.

## Introduction

The phenomenon of global population aging is gaining increasing attention due to its accelerating pace (1). As of 2019, the average life expectancy reached nearly 73.4 years, and forecasts indicate that by 2031, reaching the age of 65 will be a common milestone for people around the world. This demographic evolution is expected to result in nearly a doubling of the population aged 60 and above (2, 3). The implications of this trend are far-reaching, notably a projected surge in chronic illnesses and cognitive decline, which will heighten disability rates and the need for long-term care globally (4, 5). These developments are often accompanied by diminished physical functionality and executive cognitive deficits, leading to significant health and societal challenges among older individuals (6).

Suicide remains a major societal concern, particularly among older adults, who account for nearly 27.2% of suicide-related deaths globally (7). In the Iranian context, a comprehensive meta-analysis of 20 studies—encompassing over 271,000 suicide attempts and 22,780 fatalities—estimated the national suicide death rate at 8.14 per 100,000 individuals, with men exhibiting a higher rate (9.1 per 100,000) than women (5.0 per 100,000). Specifically, in 2023, the suicide rate among older adults in Iran was reported at 5.06 per 100,000 (8). These figures reflect an urgent public health issue, underscoring the necessity for focused psychological and social interventions targeting the older population.

One of the most potent psychological indicators of suicide risk is hopelessness (9). This condition reflects a deeply ingrained belief system characterized by persistent negative expectations regarding future events, which may drive individuals toward suicidal ideation or behavior (10, 11). As such, preventive efforts should aim to reinforce psychological resilience through improvements in general well-being, the promotion of optimism and mental flexibility, development of coping strategies, and the enhancement of social support networks—while also ensuring timely intervention for mental health disorders (12).

Among non-pharmacological approaches, physical exercise has demonstrated substantial efficacy. A synthesis of findings from 28 randomized controlled trials reveals that engaging in regular physical activity yields significant therapeutic benefits for individuals with depressive symptoms (13). Through its positive effects on both physical and mental health, physical exercise is associated with reduced rates of suicide-related behaviors (14, 15). Consequently, it is now widely endorsed in clinical practice guidelines as a key component in the treatment of psychiatric disorders and as a strategy for prolonging life expectancy and lowering suicide risks (16, 17).

Recent research further supports this perspective. A systematic review of randomized controlled trials indicated that exercise-based interventions significantly decreased suicide attempts in patients with psychological or physical disorders. Similarly, another review found that consistent physical activity among older population individuals not only reduced suicidal ideation and self-harming tendencies but also alleviated symptoms of anxiety and depression (18). Nevertheless, promoting sports participation among older adults remains a complex challenge, often hindered by factors such as low motivation and limited patience (11). Many individuals in this age group are reluctant to engage in traditional forms of physical activity (19), an issue that has received limited attention in existing research. Although physical activity has been identified as a protective factor against suicidal thoughts in older adults, there is a noticeable lack of structured programs designed to facilitate their sustained involvement. Therefore, there is an urgent need to design and implement diverse engagement strategies tailored specifically for older populations to fill this important research and practice gap.

The present study seeks to explore the effectiveness of a gamified sports system within a virtual reality (VR) environment to enhance participation in physical activity among older individuals, particularly those experiencing suicidal ideation. By integrating game-based features such as reward systems, competition, excitement, and enjoyment, the VR setting aims to foster intrinsic motivation and make sports activities more appealing and accessible to this vulnerable population (20). This quasi-experimental study employed a pre-test–post-test control group design, involving older adults with suicidal tendencies. Participants in the experimental group engaged in gamified physical exercises within a VR setting, while those in the control group did not receive the intervention. The comparative analysis of outcomes between the two groups demonstrated the effectiveness of this immersive approach as a novel intervention for addressing mental health challenges among older individuals in Iran.

Gamification refers to the incorporation of game-like mechanics and dynamics into non-gaming contexts to stimulate motivation, engagement, and attention (21). The integration of physical activity with immersive technologies such as VR and gamified elements appears particularly promising, especially for promoting exercise in home-based settings. Sports-oriented video games, often referred to as exergames, have shown potential in improving both psychological and physical well-being in older adults (22). Prior studies with active older population have confirmed that such digital games, when designed with appropriate challenge levels and feedback mechanisms, can significantly enhance user engagement and compliance (11, 23).

Virtual reality itself is a simulated digital environment that allows users to interact with computer-generated surroundings using sensory modalities such as visual, auditory, and tactile inputs, resulting in a deeply immersive experience (24). Devices like head-mounted displays, motion sensors, and interactive systems—such as the Nintendo Wii or Microsoft Kinect—are commonly used to deliver VR experiences (24). Unlike conventional gaming platforms, VR offers a fully enveloping interface that isolates the user from the real world and transports them into a highly interactive digital space (25). Despite growing interest in the use of VR and gamification in education and healthcare settings (23, 26), empirical studies investigating their application in the context of physical activity among older adults with suicidal ideation remain scarce.

This research addresses this gap by proposing a non-pharmacological, engaging intervention that combines the immersive features of virtual reality with the motivational benefits of gamified physical exercise. By tailoring the experience to the preferences and limitations of older participants, the intervention seeks to enhance their willingness to engage in regular physical activity. Ultimately, this approach is expected to improve mood, increase hope and life satisfaction, and reduce symptoms of depression and suicidal thinking. The outcomes of this study may inform future interventions in Iran and similar settings, offering innovative pathways to support mental health and active aging in high-risk older populations.

## Materials and methods

### Type of research and statistical population

This study employed a quasi-experimental, non-equivalent control group pretest–posttest design to assess the effects of a gamified virtual-reality (VR) sports intervention on life-expectancy perceptions among older men with suicidal ideation. Accordingly, the research was guided by the following hypothesis:

*H1*: Gamified sports exercises in a virtual-reality context positively influence the life expectancy perceptions of older individuals with suicidal thoughts.

While randomized experimental allocation is generally preferred to maximize internal validity, randomization was not feasible in this clinical and community-based context for practical and ethical reasons explained below. Consequently, participants were assigned to intervention and control groups using a convenience allocation procedure (non-random assignment) (27). To minimize potential bias and enhance the transparency and credibility of the findings, rigorous procedural controls were implemented throughout the study.

### Participants and setting

The target population comprised community-dwelling older men identified as experiencing suicidal thoughts. Recruitment was conducted through advertisements and clinician referrals at older care centers in Tehran, Iran. Eligibility was determined via clinical screening by geriatric and mental-health specialists and by reviewing participants’ clinical records.

Inclusion criteria were: male sex, age ≥58 years, self-reported suicidal ideation confirmed by clinical interview, ability to provide informed consent, and medical clearance to engage in low-to-moderate physical activity. Exclusion criteria included: active psychosis, recent severe cardiovascular events, significant cognitive impairment precluding participation, or concurrent involvement in other intensive exercise or psychotherapeutic interventions that could confound outcomes. In this regard, a total of 125 individuals initially expressed interest. After clinical screening and consideration of practical factors (e.g., travel distance, ongoing therapy schedules, or withdrawal of consent), 110 eligible participants were retained and allocated equally into two groups (intervention, *n* = 55; control, *n* = 55) using convenience allocation based on participant availability and safety monitoring needs.

### Sampling strategy and generalizability considerations

We acknowledge that recruitment via advertisements and clinician referrals, combined with a convenience allocation procedure, introduces potential selection bias and limits external validity. Such sampling approaches are common in geriatric and nursing-home research, where participants often differ from general older populations in motivation, access to care, and familiarity with digital technologies. These factors may enhance adherence and responsiveness to VR-based interventions. Specifically, our participants were community-dwelling, service-engaged older men who were recruited from older care centers and outpatient clinics, rather than residents of long-term care institutions or completely isolated older adults living alone. Therefore, the findings should be interpreted as most relevant to this subgroup of high-risk yet socially connected older men, and not generalized to institutionalized or homebound elders without further empirical validation.

To mitigate these limitations, the study incorporated the following measures:

- Recruitment from multiple older care centers across different districts of Tehran to enhance sociodemographic diversity.
- Standared clinical screening by geriatric/mental-health specialists to ensure consistent inclusion criteria.
- Collection of detailed baseline demographic and clinical variables and assessment of baseline equivalence between groups.
- Use of ANCOVA controlling for pretest scores and prespecified covariates, along with sensitivity analyses to confirm robustness.

Nevertheless, given the non-randomized, convenience-based design, the findings should be interpreted as relevant primarily to service-engaged, high-risk older men with suicidal ideation, rather than as directly generalizable to broader or culturally distinct populations. Future studies employing randomized, community-based sampling are recommended to strengthen external validity.

#### Rationale for non-random allocation and ethical considerations

Random allocation was precluded due to a combination of ethical and logistical constraints: (1) Many participants depended on family caregivers for transportation and scheduling, making random scheduling impractical; (2) several eligible individuals were concurrently undergoing therapy or medication management, where clinicians prioritized treatment continuity; (3) safety monitoring and caregiver coordination for this high-risk population required flexible scheduling incompatible with strict randomization.

The study was conducted under the supervision of an institutional ethics committee and in full accordance with ethical standards for human research, including informed consent, confidentiality protection, and respect for participant autonomy.

### Assessment framework and test administration

To assess life expectancy, the study utilized a modified version of the life expectancy questionnaire originally developed by Robbins (28). Given the older population’s lower morale and patience, it was important to use a single-component questionnaire that was brief yet effective in assessing life expectancy. The instrument included 12 items and was evaluated by experts in psychology to ensure its appropriateness for assessing life expectancy. To verify the scale’s reliability, internal consistency was assessed using Cronbach’s alpha, resulting in a coefficient of 0.88, which reflects a high level of reliability. This questionnaire was administered to participants in both the control and experimental groups to collect initial data on their perceptions of life expectancy before the intervention began. Participants in the experimental group underwent sports training sessions conducted in a virtual reality environment, while those in the control group engaged in traditional physical exercises without the use of VR technology. This experimental design enabled the researchers to compare pre- and post-intervention outcomes between the groups and evaluate the impact of VR-based exercise on life expectancy.

It should be noted that suicidal ideation served as an inclusion criterion and was confirmed at screening through structured clinical interviews conducted by specialist geriatric and mental-health physicians. Clinical interviews documented presence of passive or active death wishes, frequency and duration of suicidal thoughts, presence of a plan or intent, and any history of self-harm; based on these data clinicians rated each participant’s suicide risk category (low/moderate/high), which was recorded at baseline and at post-intervention. The primary quantitative outcome of the trial was perceived life-expectancy (adapted from Schneider et al.), selected because constructs related to hope/expectancy have robust empirical links to suicidal ideation and behavior and are responsive to psychosocial and exercise-based interventions. Secondary, exploratory analyses used the clinician-rated suicide-risk categories (pre vs. post) to examine whether changes in perceived life-expectancy co-occurred with clinically assessed suicidality. Changes in clinician-rated categories were analyzed with McNemar’s test for paired categorical change and with ordinal logistic regression adjusted for baseline covariates. Any documented safety events (e.g., urgent referrals, self-harm incidents) during the study period are reported descriptively.

#### Control of potential confounding variables

To ensure that the observed effects on perceived life expectancy were not confounded by pre-existing clinical or psychosocial differences, several baseline characteristics were documented and statistically controlled. During clinical screening, information on each participant’s comorbid medical conditions (e.g., cardiovascular disease, diabetes), medication use (particularly antidepressants and anxiolytics), and depressive symptom severity was systematically collected by the mental-health and geriatric specialists. Individuals presenting with severe or unstable psychiatric disorders (e.g., major depression, psychosis, or advanced cognitive impairment) were excluded from participation. Furthermore, participants were asked about their engagement in psychosocial support programs (e.g., counseling sessions, therapy groups, or social activities) during the previous 6 months. These data were used descriptively and served as covariates in subsequent statistical analyses to adjust for potential baseline imbalances. By accounting for depressive symptoms, medication status, comorbidities, and psychosocial support exposure, the study sought to isolate the independent contribution of the gamified VR intervention to improvements in life.

### Statistical analysis

To assess the distribution of the variables, the Kolmogorov–Smirnov test was applied, and all resulting *p*-values exceeded the 0.05 threshold, supporting the assumption of normality across measured components. Following this, Levene’s test was utilized to determine whether the variances between the experimental and control groups were statistically equivalent. Consequently, Analysis of Covariance (ANCOVA) was applied to account for baseline differences and ensure a robust analysis of the data.

### Steps for developing the exercise protocol

To implement sports exercises integrated with gamification within a virtual reality context, we sought the expertise and guidance of three professors and researchers specializing in sports science, gamification, and virtual reality games. Initially, a face-to-face meeting was conducted with the families and caregivers of the older participants to explain the entire research process. This approach aimed to maximize their support in fostering motivation and encouraging their relatives to fully engage in the study. Considering the specific needs and potential cognitive limitations of the older participants, a trained psychologist was present during the administration of the questionnaires to facilitate understanding and ensure the accuracy of responses.

The intervention consisted of a total of 15 gamified physical activity sessions delivered across 5 weeks. Participants attended three sessions per week, with each session lasting approximately 30–45 min depending on individual engagement levels. Key gamification elements included rewards, competition, and ranking. Older participants’ scores were recorded and compared with previous sessions, motivating them to improve their performance. Rewards were tailored to individual preferences and typically included favored foods, requests for movies, or outings for walks. Competition was fostered by comparing participants’ scores and encouraging them to surpass their peers’ performance, which positively influenced their engagement and motivation.

During the intervention, participants utilized Meta Quest VR headsets (version 3 with 512 GB of memory) to engage in gamified sports training. To maintain engagement and prevent monotony, a variety of three different games were employed. Throughout the training process, an expert in gamification and a sports specialist were present to support and guide the participants.

### Control condition

Participants allocated to the control condition received a structured traditional exercise program that was explicitly designed to match the experimental protocol in contact time, session frequency, session duration, supervision intensity, and safety monitoring. The control program comprised 15 sessions across 5 weeks (three sessions per week), with each session lasting 30–45 min — identical to the experimental arm in scheduling. Sessions were delivered in small supervised groups at the same facilities used for the VR intervention to control for setting effects. In this regards, each control session followed a standardized, progressive format: (1) a 5–10 min warm-up (gentle range-of-motion and light aerobic movements such as marching on the spot and shoulder/hip mobilization); (2) a 15–25 min main phase combining low-to-moderate intensity aerobic exercises (e.g., walking in place, step-ups onto a low platform, tandem walking), basic resistance/bodyweight movements adapted for older adults (e.g., sit-to-stand, chair-assisted squats, seated arm raises), and balance/coordination drills (e.g., tandem stance, single-leg support with hand support as needed); and (3) a 5–10 min cool-down and stretching segment. Exercise intensity was maintained at a perceived exertion of approximately 9–12 on the Borg 6–20 scale (light-to-somewhat hard), consistent with medical clearance for older adults.

The control sessions were led by the same type of trained personnel who supervised the VR sessions (a sports specialist and an exercise therapist), thereby equating instructor expertise and social interaction. Safety procedures identical to the intervention arm were applied: pre-session health checks, immediate access to on-site clinical staff, and documentation of any adverse events or urgent referrals. Adherence in the control group was recorded using session attendance logs and simple performance metrics (e.g., number of chair-stands completed in 30 s), collected to allow parity in compliance monitoring with the VR arm. To reduce motivational confounding, general positive reinforcement (praise, encouragement) was provided to control participants; however, no gamification elements (points, leaderboards, or in-session rewards) were used in the control condition.

Table 1 provides demographic information about the older, including their gender and age.

**Table 1:** Summary of participants’ demographic characteristics.

Variable	Type	Frequency	Percentage
Gender	Male	110	100%
	Female	0	0%
Age	Between 58 and 62 years	33	30%
	Between 63 and 67 years	56	50.9%
	Over 68 years	21	19.0%

## Results

As shown in Table 2, the results of the Kolmogorov–Smirnov test indicate that all study variables followed a normal distribution pattern. The reported test statistics, including the significance correction (c) and the lower bound of the true significance (d), further substantiate this finding. Consequently, it can be asserted with 95% confidence that the research variables adhere to a normal distribution, justifying the application of parametric statistical tests for hypothesis analysis.

**Table 2:** Kolmogorov–Smirnov test.

Group			Pre_suicidal	Post_suicidal
Control	*N*		55	55
	Normal parameters^a,b^	Mean	24.9636	25.8182
		Std. deviation	2.32509	2.61825
	Test statistic		0.148	0.144
	Asymp. Sig. (2-tailed)		0.004^c^	0.006^c^
Experimental	*N*		55	55
	Normal parameters^a,b^	Mean	24.2182	37.1273
		Std. deviation	2.35459	2.84185
	Test statistic		0.173	0.125
	Asymp. Sig. (2-tailed)		0.001^c^	0.032^c^

a = Tested against a normal distribution.

b = Lilliefors significance correction.

c = Including the significance correction.

Table 3 provides the main descriptive indicator relevant to the research.

**Table 3:** Descriptive indicators of research.

Group		*N*	Minimum	Maximum	Mean	Std. deviation	Variance
Control	Pre_suicidal	55	20.00	33.00	24.9636	2.32509	5.406
	Post_suicidal	55	20.00	32.00	25.8182	2.61825	6.855
	Valid *N* (listwise)	55					
Experimental	Pre_suicidal	55	19.00	32.00	24.2182	2.35459	5.544
	Post_suicidal	55	32.00	45.00	37.1273	2.84185	8.076
	Valid *N* (listwise)	55					

The outcome of Levene’s test confirmed that the variance across groups was statistically equivalent, as the *p*-value exceeded 0.05, thereby fulfilling the assumption of homogeneity. With this and other preliminary assumptions met, the dataset is deemed appropriate for conducting analysis of covariance (ANCOVA). This ensures a robust evaluation of the difference in the dependent variable between the two groups (Table 4).

**Table 4:** Analysis of variance homogeneity using Levene’s test.

*F*	df1	df2	Sig.
1.320	1	108	0.253

Referring to the group-related data summarized in Table 5, the VR-based gamified exercise intervention had a significant and substantial impact on enhancing perceived life expectancy among older individuals with suicidal ideation. A Type III Sum of Squares value of 3758.768 was observed for the group factor, with an *F* value of 796.466 (*p* < 0.001) alongside a substantial partial eta squared (*η*^2^) of 0.882. This indicates that 88.2% of the variance in perceived life expectancy can be attributed to the intervention, underscoring its effectiveness in improving the participants’ future orientation and subjective sense of hopefulness.

**Table 5:** Results of analysis of covariance (ANCOVA).

Source	Type III sum of squares	df	Mean square	F	Sig.	Partial eta squared
Corrected model	3818.452	2	1909.226	404.556	0.001	0.883
Intercept	187.758	1	187.758	39.785	0.001	0.271
Pre_suicidal	301.325	1	301.325	63.849	0.001	0.374
Group	3758.768	1	3758.768	796.466	0.001	0.882
Error	504.966	107	4.719			
Total	113282.000	110				

**Figure 1:**
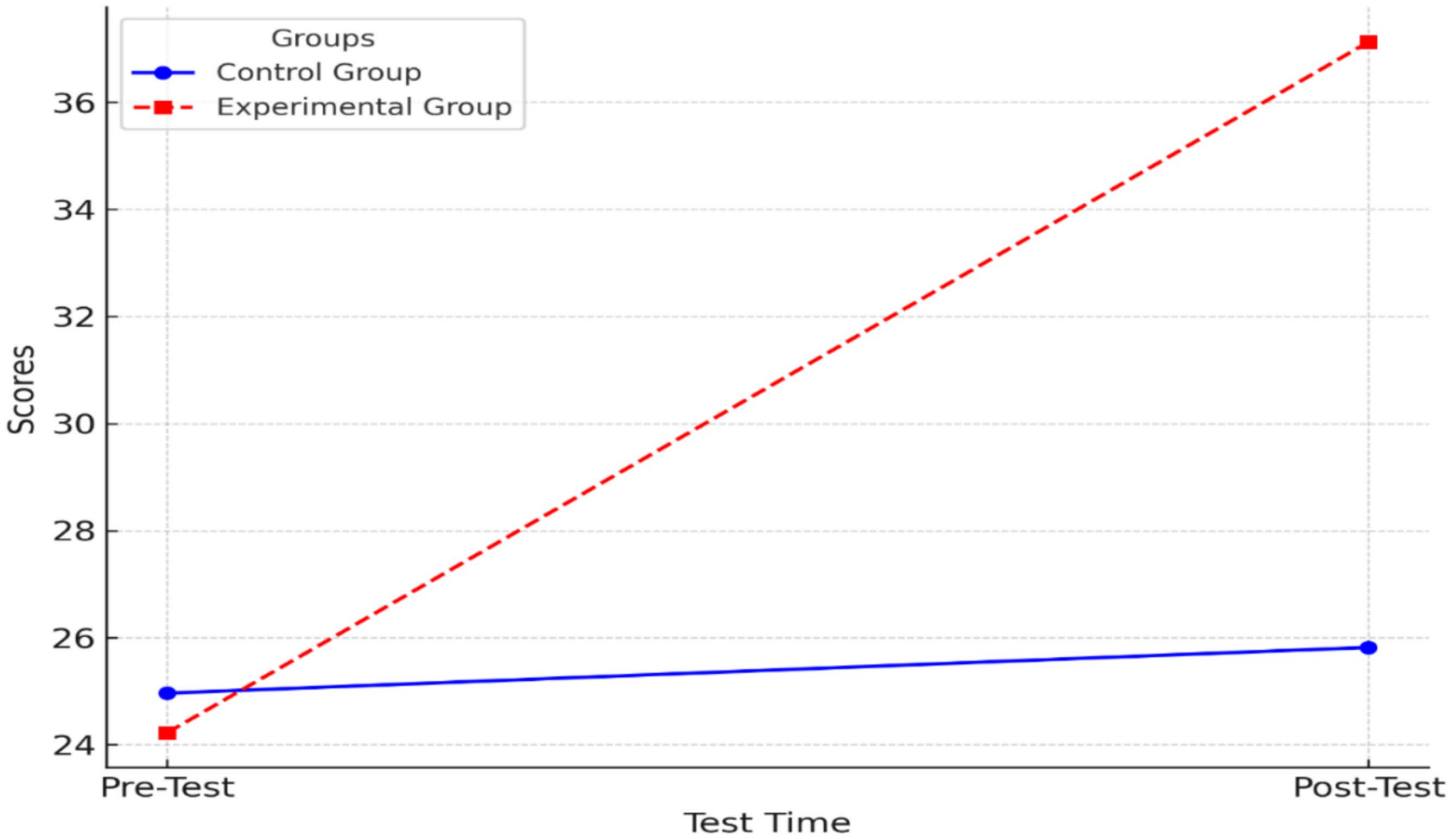
Line chart comparing mean scores.

In Figure 1, a line chart is presented to illustrate the distinction between the control and experimental groups was clarified through comparison of their mean scores in both the pre-test and post-test phases.

## Discussion

This study explored whether a gamified virtual reality (VR)–based sports program could enhance perceived life-expectancy among older men exhibiting suicidal ideation. The results revealed that participants exposed to VR-gamified exercises experienced a significant and meaningful increase in perceived life-expectancy compared with those participating in structured traditional physical exercises. These findings highlight the potential of immersive and motivationally enhanced physical activity to strengthen psychological constructs that are associated with suicidal vulnerability in late adulthood.

Physical activity is widely recognized as an effective non-pharmacological approach for improving mental health and reducing depressive symptoms (29). Previous studies have demonstrated that regular exercise contributes to greater optimism, hope, and life satisfaction in older populations (30). Similarly, interventions combining physical activity with structured psychosocial elements have been shown to enhance emotional well-being, future orientation, and motivation among older participants (18, 31). Our findings are consistent with this literature and suggest that VR-based exercise, through its immersive and interactive environment, can deliver additional motivational benefits beyond those observed with conventional exercise. Consistent with Goumopoulos et al. (32), VR technologies can support engagement and adherence among older adults by providing immediate feedback and interactive goal structures (20). Also, in a 2025 study, Dong et al. investigated the effects of virtual reality-based physical activity for vulnerable older populations, that aligns with our study’s results. Their findings demonstrated that this intervention can lead to statistically significant improvements in subjective well-being. In this regard, elements embedded in our intervention—such as point scoring, progress tracking, and adaptive challenges—likely enhanced engagement and emotional arousal, reinforcing the psychological impact of physical activity (11). This interpretation aligns with recent systematic reviews and meta-analyses indicating that gamified health interventions yield modest to moderate improvements in adherence and affective outcomes (33, 34). The mechanisms plausibly underlying observed changes are likely multifactorial. Enhanced motivation arising from gamification fosters active participation, which may mediate gains in perceived control and self-efficacy (35). Immersion and novelty effects inherent to VR reduce the perception of monotony and perceived exertion, promoting sustained engagement (36). Physiological pathways of exercise (e.g., endorphin release, improved sleep, and autonomic regulation) can improve mood and cognitive functioning, while supervised group sessions provide social contact and professional reinforcement that reduce isolation (37). Collectively, these psychosocial and biological processes can contribute to improved future orientation and well-being.

Importantly, we conceptualized perceived life expectancy as a proxy indicator of suicidal vulnerability rather than a direct measure of suicidal ideation. This conceptualization aligns with prior evidence showing that shortened future time perspective and diminished life expectancy appraisals are robust correlates of hopelessness, a well-established cognitive precursor of suicidal behavior (38, 39). Moreover, recent studies have demonstrated that interventions enhancing future-oriented cognition and perceived control over aging can indirectly mitigate suicide risk among older adults by reducing feelings of entrapment and purposelessness (40). Accordingly, improvements in perceived life expectancy in our study may reflect a broader enhancement of psychological resilience and reduction in cognitive vulnerability factors linked to suicide risk, although such an interpretation should remain cautious given that we did not employ a dedicated self-report suicidality instrument in this protocol. Instead, clinician-rated suicidality categories were used for exploratory analyses, which provide supportive but not definitive evidence for this association. In this regard, our results are in line with meta-analytic evidence indicating that VR-exergame interventions can yield beneficial effects on mood, cognitive function, and quality of life in older adults. These syntheses support the plausibility that technology-assisted physical activity can address both physical and psychological dimensions of healthy aging, particularly in populations who show low adherence to conventional exercise programs.

Participant safety and ethical integrity were treated as central priorities throughout the intervention, particularly given the vulnerability of older adults with elevated suicide risk. All individuals underwent thorough clinical screening before participation, and sessions were continuously supervised by a licensed clinical psychologist and a geriatric exercise specialist. A structured safety protocol—comprising real-time observation, immediate psychological support, and post-session debriefing—was implemented to mitigate potential distress associated with VR immersion. Minor side effects such as transient dizziness or lightheadedness were reported by approximately 10% of participants and resolved rapidly without clinical intervention; no serious adverse or crisis events occurred. Consistent with these, Wang et al. observed that dizziness subsided in men receiving the virtual reality intervention after approximately 5–10 min (23). These findings are consistent with prior work indicating that, under controlled conditions, VR-based exercise can be safely administered in older adults, including those with mild psychological vulnerability (41, 42). Beyond safety, the psychosocial engagement facilitated by the gamified VR environment appears to have contributed meaningfully to participants’ cognitive and emotional well-being (21). The combination of immersive presence, goal-directed challenges, and real-time feedback likely enhanced motivation, agency, and perceived control—mechanisms repeatedly linked to improved mood and future orientation in older adults (43). Similarly, Faridniya and George found that the combined mechanism of gamification and general exercises in virtual reality also led to enhanced motivation and a reduction in cognitive dissonance among participants (44). In this sense, the observed improvements in perceived life expectancy may reflect an adaptive shift in cognitive appraisal of the future, rather than a direct reduction in suicidal ideation. This interpretation aligns with prior theoretical and empirical evidence suggesting that enhanced future time perspective and perceived purpose are protective against suicidal vulnerability (45, 46).

It is also important to consider the potential influence of the Hawthorne Effect when interpreting these findings. Participants in the VR intervention group may have exhibited enhanced motivation or optimism simply because they received more attention from instructors and perceived themselves as part of an innovative technological program. This increased engagement, stemming from heightened awareness of being observed or supported, could have contributed to short-term improvements in perceived life expectancy. However, given that both groups were provided with comparable levels of social interaction and professional supervision, the magnitude of this effect is likely limited. Future studies employing blinded assessment protocols or longer follow-up periods could help clarify the extent to which observed benefits reflect true intervention effects rather than reactivity to observation. Overall, the results indicate that gamified VR-based exercise can be feasibly and safely implemented as a complementary strategy within supervised care settings to promote psychological resilience and a more hopeful orientation toward the future among at-risk older adults. Nevertheless, any clinical application should be accompanied by rigorous safety monitoring, professional oversight, and ongoing evaluation of ethical and psychological implications to ensure that such technologies remain both effective and humane in this sensitive population.

### Limitations and future research

This study has several limitations that warrant consideration. First, outcome data were collected immediately after the five-week intervention, which restricts our ability to determine the long-term persistence of the observed improvements in perceived life expectancy. It remains unclear whether these gains would be sustained over months or gradually diminish once the structured VR sessions are discontinued. Future studies should incorporate follow-up assessments at multiple time points (e.g., 3 or 6 months post-intervention) to evaluate the durability of effects and the potential need for booster sessions. Second, while our sample included older men with clinically identified suicidal ideation, the findings cannot be generalized to broader older adult populations or to women without further validation. Expanding research to more diverse demographic groups and clinical contexts would enhance the external validity of the results. Third, although the current design allowed for strong internal control, it relied on clinician-rated measures of suicidality rather than direct self-report scales. Future work could benefit from including multi-method assessments—combining psychometric, behavioral, and physiological indicators—to obtain a more comprehensive understanding of psychological changes associated with VR-based interventions. Finally, because participants were community-dwelling older men already connected to older care centers and clinical services, the results may not be directly generalizable to all older adults—particularly those residing in long-term care institutions or living completely independently without structured social or medical support. The psychosocial and environmental contexts of these groups can differ substantially, potentially moderating the effectiveness of gamified VR-based exercise interventions. Therefore, future research should aim to replicate and extend these findings among more diverse older populations to determine the generalizability and contextual boundaries of such interventions.

## Conclusion

This investigation assessed the contribution of gamified virtual reality training to changes in life expectancy among older individuals with suicidal thoughts. The findings indicated that this innovative approach significantly enhances both physical and mental well-being. Participants engaging in VR programs showed improved life expectancy and reduced levels of depression and anxiety. The immersive nature of VR gamification effectively increased motivation and adherence to physical activities, addressing the challenges older individuals often face with traditional exercise programs. Additionally, the social interaction and goal-setting elements in gamified VR environments foster a sense of achievement and connection, alleviating feelings of isolation and hopelessness. These combined benefits contribute to overall health improvement and increased life expectancy. Incorporating VR-based gamified exercise programs into treatment regimens could significantly enhance the mental and physical health of older individuals with suicidal thoughts. Subsequent studies are encouraged to assess the sustainability and accessibility of such interventions among varied older demographics.

## Data Availability

The raw data supporting the conclusions of this article will be made available by the authors, without undue reservation.
